# Laminin modification subretinal bio-scaffold remodels retinal pigment epithelium-driven microenvironment *in vitro* and *in vivo*

**DOI:** 10.18632/oncotarget.11502

**Published:** 2016-08-22

**Authors:** Chi-Hsien Peng, Jen-Hua Chuang, Mong-Lien Wang, Yong-Yu Jhan, Ke-Hung Chien, Yu-Chien Chung, Kuo-Hsuan Hung, Chia-Ching Chang, Chao-Kuei Lee, Wei-Lien Tseng, De-Kuang Hwang, Chia-Hsien Hsu, Tai-Chi Lin, Shih-Hwa Chiou, Shih-Jen Chen

**Affiliations:** ^1^ Department of Ophthalmology, Taipei Veterans General Hospital, Taipei, Taiwan; ^2^ Department of Medical Research, Taipei Veterans General Hospital, Taipei, Taiwan; ^3^ Department of Ophthalmology, Shin Kong Wu Ho-Su Memorial Hospital & Fu-Jen Catholic University, Taipei Taiwan; ^4^ Department of Ophthalmology, Tri-Service General Hospital & National Defense Medical Center, Taipei, Taiwan; ^5^ Department of Biological Science and Technology, National Chiao Tung University, Hsinchu, Taipei, Taiwan; ^6^ Department of Photonics, National Sun Yat-sen University, Kaohsiung, Taiwan; ^7^ National Health Research Institute, Hsinchu, Taiwan; ^8^ Institute of Clinical Medicine, National Yang-Ming University, Taipei, Taiwan; ^9^ Institute of Pharmacology, National Yang-Ming University, Taipei, Taiwan; ^10^ School of Medicine, National Yang-Ming University, Taipei, Taiwan

**Keywords:** age-related macular degeneration, biomimetic scaffold, pluripotent stem cells, pigment epithelium cells, pigment epithelium-derived factor, Pathology Section

## Abstract

Advanced age-related macular degeneration (AMD) may lead to geographic atrophy or fibrovascular scar at macular, dysfunctional retinal microenvironment, and cause profound visual loss. Recent clinical trials have implied the potential application of pluripotent cell-differentiated retinal pigment epithelial cells (dRPEs) and membranous scaffolds implantation in repairing the degenerated retina in AMD. However, the efficacy of implanted membrane in immobilization and supporting the viability and functions of dRPEs, as well as maintaining the retinal microenvironment is still unclear. Herein we generated a biomimetic scaffold mimicking subretinal Bruch's basement from plasma modified polydimethylsiloxane (PDMS) sheet with laminin coating (PDMS-PmL), and investigated its potential functions to provide a subretinal environment for dRPE-monolayer grown on it. Firstly, compared to non-modified PDMS, PDMS-PmL enhanced the attachment, proliferation, polarization, and maturation of dRPEs. Second, PDMS-PmL increased the polarized tight junction, PEDF secretion, melanosome pigment deposit, and phagocytotic-ability of dRPEs. Third, PDMS-PmL was able to carry a dRPEs/photoreceptor-precursors multilayer retina tissue. Finally, the *in vivo* subretinal implantation of PDMS-PmL in porcine eyes showed well-biocompatibility up to 2-year follow-up. Notably, multifocal ERGs at 2-year follow-up revealed well preservation of macular function in PDMS-PmL, but not PDMS, transplanted porcine eyes. Trophic PEDF secretion of macular retina in PDMS-PmL group was also maintained to preserve retinal microenvironment in PDMS-PmL eyes at 2 year. Taken together, these data indicated that PDMS-PmL is able to sustain the physiological morphology and functions of polarized RPE monolayer, suggesting its potential of rescuing macular degeneration *in vivo*.

## INTRODUCTION

Age-related macular degeneration (AMD) is a worldwide leading cause of blindness especially in developing countries [1]. Patients with end-stage AMD lost their central vision permanently mainly due to fibrovascular scar or atrophy of retinal pigment epithelium (RPE) and photoreceptors in macula [1, 2]. Current treatments focused on controlling growth and leakage of choroidal neovessels in wet-type AMD by injecting anti-vascular endothelial growth factor (VEGF) repeatedly [3]. The visual outcomes were usually restricted due to persistence of the fibrous tissue and the loss of RPE and photoreceptors [1, 4]. Moreover, recent studies showed that the maintenance of subretinal environment is crucial for functional RPE; dysregulated subretinal environment may be involved in the pathogenesis of AMD, as well as the key to treat AMD progression to restore the retinal function in advanced AMD. However, scarce treatments focused their effect on RPE and neurosensory retina, or reconstructing the integrity of subretinal environment, of which the dysfunction and degeneration may weaken the blood-retina-barrier and cause AMD originally.

Retinal pigment epithelium (RPE) cells are essential for the maintenance of normal physiology within the neurosensory retina and photoreceptors [1, 5]. RPE cells phagocytose the tips of photoreceptor outer segments and help to maintain the blood-retina barrier [5-8]. Because RPE cells are so essential for maintaining the integrity of the subjacent choriocapillaris and blocking the subretinal neovascularization, RPE degeneration and loss has been implicated as the key factor of the initiation and progression of AMD [2]. Given the shortage of therapeutic drugs to treat the advanced AMD, transplantation of RPE, photoreceptor, or other retinal cells is an alternative way to repair the damaged retina in AMD patients. However, obtaining a sufficient number of suitable donor RPE and photoreceptors for *ex vivo* transplantation in rescuing the visual dysfunction of AMD is still an obstacle for such therapy. Therefore, pluripotent stem cell-based therapy, such as embryonic stem cells (ESC) and induced pluripotent cells (iPSC), is a potential resolution for the limited donor's RPE in regeneration medicine [9]. Moreover, recent studies indicated that the polarized monolayer of RPE showed better survival and growth compared with suspended RPE cells [10]. Transplanting pluripotent stem cell-differentiated RPE as a sheet of monolayer has more potential for a successful retinal repair, particularly for the geographic atrophy in dry-AMD patients that need to repair a rather large area of retina [11]. However, the biosafety and efficacy of the transplantable materials, as well as the visual-functional improvement of the implanted RPE cells in the subretinal space remain to be determined.

Bruch's membrane is a 2- to 4-μm-thick acellular, ultrathin tissue located between the retina and choroid. Bruch's membrane is abundant in collagen I and IV, laminin, fibronectin, elastin, and lipoprotein [12-14]. These components attribute to the elasticity of Bruch's membrane and transportation of nutrients and wastes to and from the retina [15, 16]. The purpose of the current study is to develop a Bruch's membrane-like biomimetic scaffold that can facilitate the growth and promote the functions of human pluripotent stem cell-differentiated RPE cells (dRPE) implanted in the subretinal space *in vivo.* Firstly, we modified polydimethylsiloxane (PDMS) with O2 plasma treatment and laminin coating (PDMS-PmL) to enhance the adherence of functional dRPE monolayer as well as photoreceptor/dRPE multilayer of retinal cells. Furthermore, we demonstrated a PDMS-PmL-based transplantable and biocompatible scaffold that can carry the polarized dRPE-monolayer and maintains RPE functions, including PEDF secretion and phagocytosis. A long-term implantation study in porcine eyes with 2-year fallow-up demonstrated the biosafety of PDMS-PmL, and its effectiveness to maintain the PEDF concentration in retinal microenvironment as well as the light response determined by multifocal ERG. Our results indicated a potential application of the O2 plasma-modified and laminin-coated PDMS sheet for functional repair in damaged retina, especially for macular degeneration.

## RESULTS

### Generation of pluripotent stem cell-derived RPE monolayer

**Figure 1:**
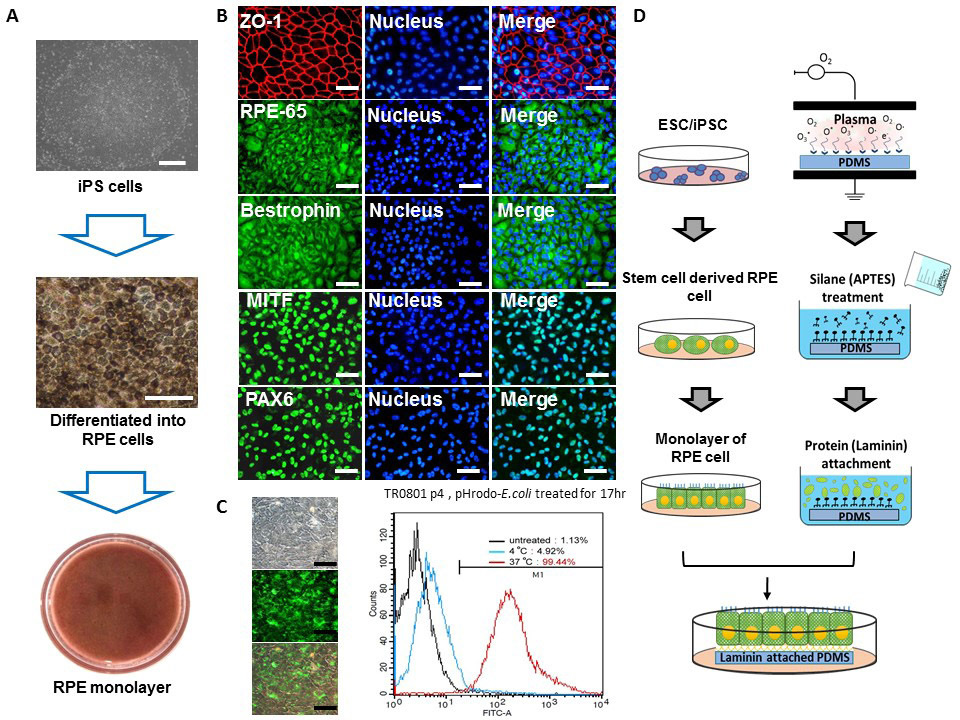
Generation of RPE monolayer from patient-specific iPS cells **A.** Microscopic photos of AMD patient-specific iPS cells, differentiated RPE cells, and PRE monolayer. Scale bar = 100 μm. **B.** Immunostaining of RPE specific protein markers, Rpe65, bestrophin, MITF, and PAX, as well as the ZO-1 tight junction marker, in dRPE cells. Scale bar = 50 μm. **C.** dRPE cells were subjected to a phagocytosis assay by incubated with pH-sensitive red fluorescence-conjugated *E. coli* particles for 18 hours. The change of fluorescence dye was observed under fluorescence microscope (left) and quantified by flow cytometry (right). Scale bar = 100 μm. **D.** Schematic illustration of the designed procedures for biomimetic subretinal implant.

Pluripotent cell-derived RPE has been used in the repair of retina disease in several animal models [17], as well as tested in pre-clinical trials for repairing the degenerated RPE in advanced AMD patients [18, 19]. We previously established human iPSC cell lines from T-cells through delivering Oct4, Sox2, Klf4, Lin28, Myc, and sh-p53 by electroporation [20, 21] (Figure 1A, top, Suppl. Information). The human iPSCs were then differentiated into RPE cells (Figure 1A, middle, Suppl. Information) for further *in vitro* and *in vivo* studies [20]. These pluripotent cell-differentiated RPE (dRPE) presented hexagonally-packed morphology with heavy pigmentation (Figure 1A, middle and bottom), and expressed RPE specific protein markers such as RPE65, bestrophin, MITF, and PAX6, as well as the Zonula occludens-1 (ZO-1), a tight junction-specific protein (Figure 1B). To examine the phagocytosis function of the dRPE cells, we incubated dRPE cells with the pH-sensitive pHrodoTM E. coli fluorescent bioparticles to visualize the engulfment of phagosomes. As shown in Figure 1C, dRPE cells expressed high level of red fluorescence, which is induced when cells undergo phagocytosis and engulf the particles in phagosomes. Quantification of the red fluorescence revealed significant enhanced phagocytotic activity in dRPE cells, compared with control (Figure 1C, right). Collectively, these analyses confirmed that dRPE cells possessed typical RPE morphology, markers expression, phagocytosis function, and tight junction of cell contact. Furthermore, physiological morphology of human RPE is a polarized monolayer lining with under the layer of photoreceptors to provide essential nutrients and engulfs the tips of photoreceptor outer segments [1, 7, 22, 23]. It is critical for RPE cells to maintain their physiological organization to improve their survival and exert their functions [10, 24]. We then designed a PDMS-based biomimetic film aiming to support the polarized dRPE monolayer for implantation in subjects' subretinal space *in vivo* (Figure 1D).

### O_2_ plasma treatment on PDMS modification with and laminin coating

**Figure 2:**
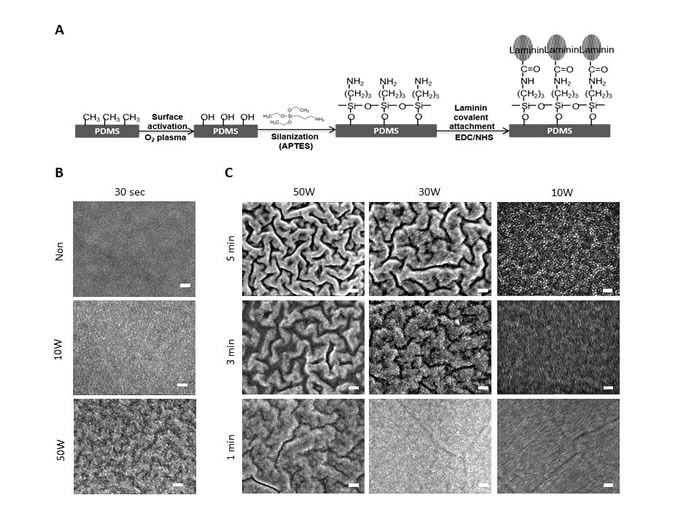
Plasma modified PDMS sheet **A.** Schematic illustration of the surface modification procedure of PDMS. PDMS membranes were generated using medical grade elastomer (Nusil Technology LLC, Inc.), and PDMS surfaces was treated with O_2_ plasma. After plasma modification, silane solution (APTES) was used to introduce the amine groups (NH_2_) onto the surface for further protein attachment. Laminin was chemically attached on to the aminated surface *via* EDC/NHS mediated covalent bonding. In the reaction, the carboxylic acid group son laminin enable to couple with the amine group on PDMS surface and finally form a stable amide bond on PDMS substrate. **B.** SEM images of PDMS substrate with (10W and 50W) or without plasma treatment for 30 sec. The samples were dried in vacuum and then sputter coated with gold (JFC 1200, JOEL Tokyo, Japan). Images were obtained using a JSM-7600F (JOEL, Tokyo, Japan) SE) with electron beam energy of 5kV. Small granular-like structure appears on PDMS surface after plasma treated at 10W. Wave structure formed on the surface after plasma treated at 50W. Scale bar = 100 nm. **C.** SEM images of plasma treated ODMS substrates with different plasma exposure time and power. Scale bar = 100 nm.

The PDMS is a popular bio-safe material with high bio-compatibility that has been widely used for microfluidic device construction, especially for biological application [25]. However, its low cell adhesiveness and high hydrophobicity causes its major drawbacks and results in substantial sample loss. In order to overcome the disadvantages of PDMS, we executed surface modification on PDMS substrates to enhance its adhesiveness and reduce its hydrophobicity. Figure 2A illustrates the surface modification scheme used to functionalize PDMS substrate. We first pre-treat PDMS surface with O_2_ plasma to introduce the OH group, as described in previous studies [26, 27]. PDMS surfaces then became hydrophilic after the O_2_ plasma treatment due to the presence of silanol (Si-OH) group on the surface. The morphology of PDMS membrane before and after 30 sec of O_2_ plasma treatment was observed by scanning electron microscopy (SEM) (Figure 2B). The plasma-treated PDMS expressed granular surface in comparison to the homogenous non-treated PDMS. The oxygen content on the PDMS surface would increase along with both the increased plasma power and prolonged exposure time; however, this may also cause etching on the surface and increase the surface roughness on PDMS substrate, which affects the subsequent cellular attachment on the surface. To evaluate the optimal condition of PDMS modification, PDMS films were treated by O_2_ plasma with serial titrations of power and different exposure times (Figure 2C). The PDMS surface were significantly etched when treated with 50W and 30W of O_2_ plasma, whatever the exposure time was (Figure 2C). When the plasma power was reduced down to 10W, the PDMS surface appeared to maintain its integrity (Figure 2C). With a serial test of low-power plasma treatment, we found the 10W/5-min treated PDMS seems to be maintaining better quantity for treating silanization and laminin-coating on PDMS as compared to others (data not shown). Hence, the 10W/5-min plasma modification condition was chosen as the standard treatment for this study.

### PDMS modification with O_2_ plasma treatment and laminin coating

**Figure 3:**
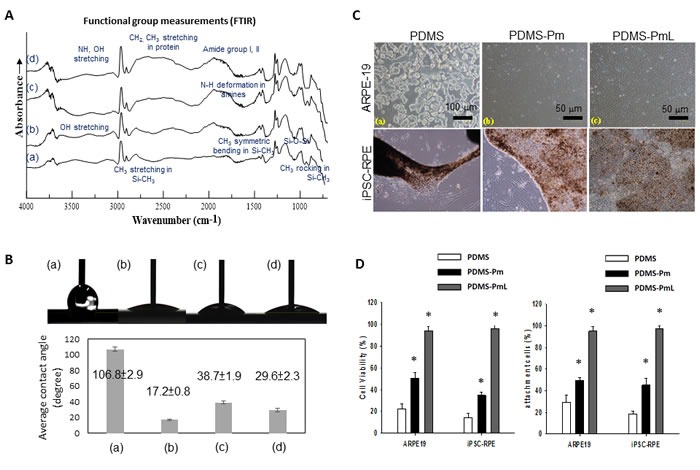
Characterization of plasma modified and laminin attached PDMS sheet **A.** The chemical composition of PDMS substrates was confirmed using a grazing angle reflectance (80^o^) FTIR measurement. The characteristic peaks of unmodified PDMS substrate include: symmetric and asymmetric -CH3 stretching from the ≡Si-CH3 group at 2870 and 2970 cm-1, respectively; symmetric -CH3 bending from the ≡Si-CH3 group at 1259 cm-1; -CH3 rocking from the ≡Si-CH3 group at 793 cm-1; Si-O-Si peaks at 1076 and 1018 cm-1. PDMS substrate with plasma treatment are increased the -OH stretching from 3100-3600 cm-1 in the IR spectra. After the reaction of protein attachment completed, the presence of protein on the surface is characterized by the peaks corresponding to the amide I (H-CO-NH2), amide II (H-CO-NHR), and amide III (H-CO-NHRR') at 1640, 1550 and 1320 cm-1, respectively. The results confirmed the coating of laminin on plasma modified PDMS surface. **B.** Water contact angles on the surfaces of a: unmodified PDMS, b: O2 plasma treated PDMS (PDMS-Pm), c: aminized PDMS-Pm, and d: PDMS-PmL were measured at ambient temperature by a video-image sessile drop tensiometer (top) and quantified in the chart (bottom). Surface roughness and heterogeneous were characterized by dynamic contact angle measurement. The results show the laminin-coated PDMS becomes more hydrophilic and increase surface roughness (reflected by the angle of contact angle hysteresis), resulting in a highly favored surface for cell attachment. **C.** iPSC-differentiated RPE and the ARPE-19 cell lines were seeded on the PDMS, 10W/5-min plasma treated PDMS (PDMS-Pm), and 10W/5-min plasma treated PDMS with laminin coating (PDMS-PmL). The morphology of cells was observed under microscope. **D.** The quantified cytotoxicity (left) and attachment (right) of dRPE and ARPE-19 on PDMS, PDMS-Pm, and PDMS-PmL films. * *P* < 0.05.

Using a grazing angle reflectance (80^o^) FTIR, the surface characteristics of unmodified, plasma treated, surface aminized, and laminin-coating/plasma-treated PDMS (PDMS-PmL) were shown in Figure 3A, and the presence of laminin on PDMS-PmL surface was proved. To assess the hydrophilic property of the modified PDMS, we measured the water contact angle on the surfaces of PDMS with different modification, and demonstrated that the PDMS-PmL presented increased hydrophilic property compared with non-modified PDMS control (Figure 3B). Moreover, we examined the effect of laminin-coating on cell adherence to PDMS. The results showed that both dRPE cells and ARPE-19 RPE cell line formed a hexagonal monolayer on PDMS-PmL but less homogenous on PDMS-Pm (O_2_ plasma only without laminin-coating), while both cells had difficulty to attach on the non-modified PDMS (Figure 3C). We then examined the cytotoxicity and attachment of dRPE and ARPE-19 on PDMS, PDMS-Pm, and PDMS-PmL. As shown in Figure 3D, both cells had better survival and highest percentage of attachment when grown on PDMS-PmL, compared with non-modified PDMS and PDMS-Pm. Taken together, these analyses showed that the modification with plasma treatment and laminin coating increased the hydrophilic property of PDMS, allowing a better growth and attachment of dRPE cells.

### Ultrastructure of the dRPE monolayer on PDMS-PmL

**Figure 4:**
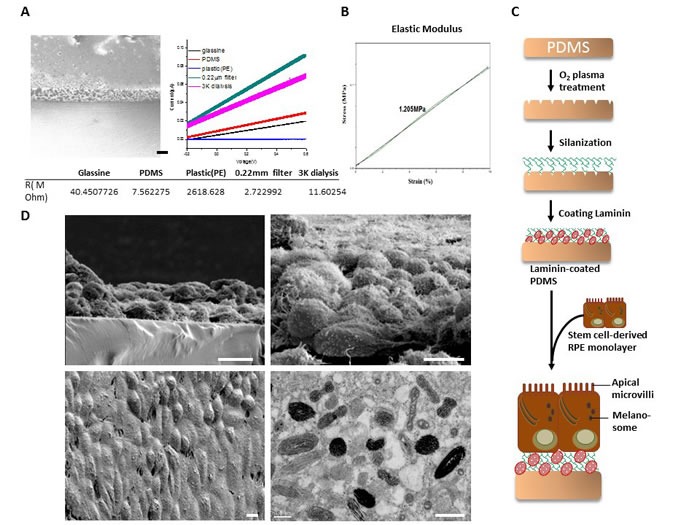
Electron microscopic structure of the dRPE monolayer on PDMS-PmL **A.** Left panel: the SEM image of porous PDMS. Scale bar = 100 μm. Right panel: The conducting current of glassine membrane (black line); current response of porous DPMS (red line); current response of plastic membrane (orange line); current response of 0.22 μm filter membrane (green line); current response of 3K cut-off dialysis membrane (blue line). The potentials in this work were measured with direct relevance to the Ag/AgCl reference electrode. **B.** The elastic modulus of porous PDMS. **C.** Schematic illustration of generating the dRPE/PDMS-PmL biomimetic film. **D.** SEM of the dRPE monolayer on PDMS-PmL film (top left, scale bar = 10 μm.) and the typical hexagonal organization of dRPE cells on PDMS-PmL (bottom left, scale bar = 10 μm.). SEM image of the apical microvilli of dRPE cells when grown on PDMS-PmL film (top right, scale bar = 10 μm.). A TEM image revealed the cellular melanosome deposit of the dRPE cells when grown on PDMS-PmL film (bottom right, scale bar = 500 nm.).

We then evaluated if this modification affect the conductance and diffusing ability of PDMS. As shown in the left panel of Figure 4A, PDMS-PmL still presents the multi-porous characteristics. By using modified electrochemical spectroscopy analysis (Suppl. Information), the conductance and diffusing ability of this porous PDMS-PmL is better than glassine and PE membrane, but the effective pore size may be smaller than 0.22 μm filter (Merk Millipore corp., Darmstadt, Germany) and 3.5K cut off dialysis membrane (Cellu.Sep, Membrane Filtration Products, InC., Seguin, TX, USA) (Figure 4A, right). These results indicate that pore size of porous PDMS-PmL is larger than sucrose molecule (∼9 Å) which can penetrate the glassine membrane [28] but smaller than 30 amino acid peptides (∼2nm) [29]. Therefore, the effective pore size of our porous PDMS-PmL is approximately from 0.9 ∼2 nm. Moreover, the result of elastic modulous examination also indicated that the modification did not affect the elasticity of PDMS (Figure 4B). Taken together, these data suggested that the small molecules, including essential-nutrients like glucose, sucrose, amino acids, and small peptides, could directly pass through PDMS-PmL to provide an implanted environment for maintaining the survival of dRPE cells.

To further examine whether PDMS-PmL facilitates the polarization of dRPE, we seeded the dRPE cells on PDMS-PmL as illustrated in Figure 4C. Using scanning electron microscope (SEM), the ultrastructural morphology of dRPE surfaces and the coating of dRPE monolayer on PDMS-PmL were visualized. The surface of PDMS-PmL appears smooth and compact with significant integrity, avoiding cells to migrate through the membrane (Figure 4D). In SEM images, the formation of a flat and polarized RPE monolayer was observed on PDMS-PmL (Figure 4D , top left). These dRPE cells also appeared to retain a uniform hexagonal shape (Figure 4D , bottom left) and abundant apical microvilli on the laminin grafted PDMS-PmL (Figure 4D , top right). Using transmission electron microscope (TEM), we showed that these dRPE cells expressed melanosome pigment deposit, a typical character of mature RPE cells (Figure 4D, bottom right). Taken together, our findings indicated that the surface of modified PDMS-PmL membrane provides a basement membrane-like environment for RPE growth and polarization with tight-layout in monolayer structure, the physiological morphology of RPE layer.

### PDMS-PmL enhanced the differentiation and functional maturation of dRPE cells

**Figure 5:**
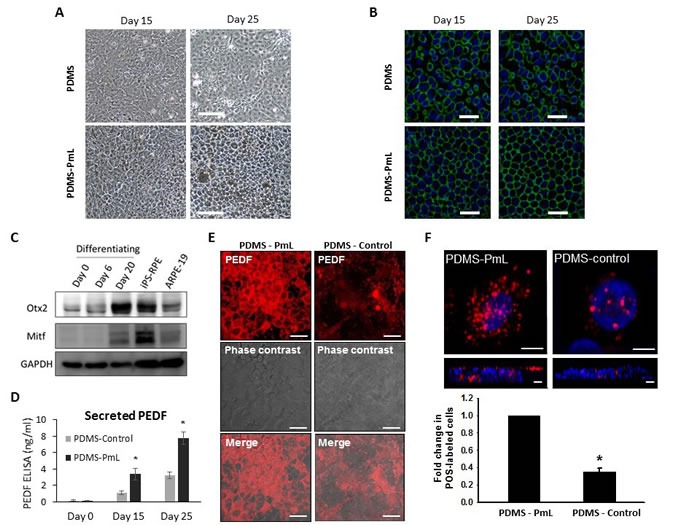
Evaluation the growth and function of dRPE cells on PDMS-PmL **A.** Patient-specific iPSC were seeded on PDMS-control and PDMS-PmL and subjected to the RPE differentiation protocol. The cell morphology at day 15 and 25 were observed under microscope. Scale bar = 100 μm. **B.** The differentiated RPE cells on PDMS-control and PDMS-PmL were subjected to an immunostaining for ZO-1. Scale bar = 50 μm. **C.** Western blot analysis of the RPE specific proteins Otx2 and Mitf in cells undergoing the RPE differentiation protocol at indicated days. **D.** dRPE/PDMS-Control and dRPE/PDMS-PmL were subjected to an ELISA assay to assess the secreted levels of PEDF. **E.** Immunofluorescent staining of PEDF on dRPE/PDMS and dRPE/PDMS-PmL biomimetic films. Scale bar = 50 μm. **F.** dRPE/PDMS and dRPE/PDMS-PmL were subjected to a phagocytosis assay by incubated with pH-sensitive red fluorescence-conjugated *E. coli* particles for 18 hours. The change of fluorescence dye was observed under fluorescence microscope. Scale bar = 25 μm.

Laminin is one of the important components of the retinal extracellular matrix, as well as the microenvironment niche for stem cell differentiation [30]. To further explore whether PDMS-PmL facilitates RPE differentiation and maturation, human iPSCs were seeded on PDMS and PDMS-PmL before undergoing RPE differentiation protocol. Observation of the dRPE cells under microscope showed that during the 25 days of differentiation, cells on PDMS-PmL presented better attachment and hexagonal organization compared with cells on PDMS (Figure 5A). Moreover, dRPE cells on PDMS-PmL expressed more melanosome pigment deposit since day 15 of the differentiation procedure (Figure 5A). Importantly, immunofluorescent staining of the tight junction-specific ZO-1 protein demonstrated a better organization of ZO-1 at the cell-cell contact of dRPE cells seeded on PDMS-PmL than that on PDMS-control from day 15 after the induction of differentiation (Figure 5B). Western blot analysis of the iPSCs under on-film RPE differentiation protocol demonstrated a gradual increased expression of the Otx2 retinal-lineage marker and the Mitf RPE-specific protein (Figure 5C). Notably, the RPE differentiation procedure, which normally takes 30 to 40 days under standard protocol, spent only 25 days on PDMS-PmL to generate mature RPE cells (Figure 5A-5B). These data suggested that PDMS-PmL may provide a niche for efficient RPE differentiation. Moreover, we further evaluate the functional maturation of the dRPE cells on PDMS-PmL by analyzing PEDF secretion level and phagocytosis capability. The ELISA analysis demonstrated an increase of secreted PEDF in the culture medium of dRPE/PDMS-PmL, compared with dRPE/PDMS-control at day 15 and day 25 post-differentiation (Figure 5D). Immunofluorescent staining of extracellular PEDF also supported that PDMS-PmL enhanced the PEDF secretion of dRPE cells (Figure 5E). In line with increased PEDF secretion, dRPE presented a better phagocytosis activity on PDMS-PmL than growing on PDMS-control (Figure 5F). All together, our molecular, morphological, and functional analyses confirmed that the modification PDMS with plasma treatment and laminin coating facilitated the differentiation and functional maturation of RPE cells, at least in stem cell-based retinal-lineage differentiation.

### PDMS-PmL is able to carry multilayers of photoreceptor precursor/RPE

**Figure 6:**
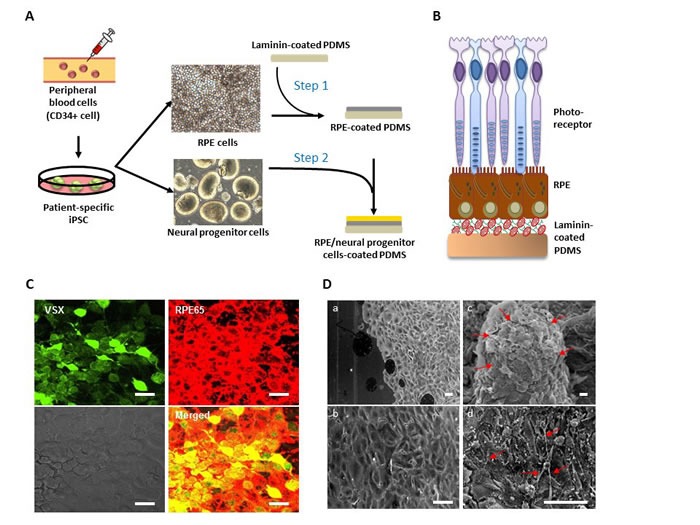
Development of PDMS-PmL carried multilayer of iPSC-derived retinal tissues **A.** Schematic illustration of the procedure for producing iPSC/neural progenitor-derived photoreceptor progenitor and dRPE bilayer-coating PDMS-PmL biomimetic film. **B.** Schematic figure of the iPSC/neural progenitor-derived photoreceptor precursor/dRPE/PDMS-PmL device. **C.** Immunofluorecent staining for neural progenitor-derived photoreceptor precursor (VSX) and dRPE (RPE65) bilayer co-cultured on PDMS-PmL film. Scale bar = 50 μm. **D.** SEM analysis of the neural progenitor-derived photoreceptor precursor/dRPE bilayer coated on PDMS-PmL biomimetic film. Scale bar = 100 μm. a: monolayer of dRPE on non-modified PDMS; b: monolayer of dRPE on the PDMS-PmL film; c: neurosphere seeded on top of the dRPE monolayer; d: the differentiated neural progenitor cells with typical spiky morphology growing on top of dRPE monolayer.

Retina is a complex combination of tissues consisted of several different layer of cells including RPE, photoreceptor, bipolar retinal nerve cells, and retinal ganglion cells. The generation of photoreceptor cells for use in conjunction with the RPE graft would be a solution for recovering the visual dysfunction in severe retinal degeneration. In an attempt to mimic physical structure of retina and examine the diverse applications of PDMS-PmL in severe retinal degeneration like late-stage AMD, we examined the capability of PDMS-PmL membrane to carry multilayer of retinal cells (Figure 6A). Elegant study by Zhong et al. reported the generation of three-dimensional retina with photoreceptor-like precursors from iPSCs [31]. Following this protocol, we produced photoreceptor precursors from iPSC (Figure 6A Bottom). Meanwhile, our previous work demonstrated the use of RPE-monolayer as a retinal cell-to-cell environment facilitates stem cell differentiation toward neural progenitor cells [32]. For constructing the iPSC-derived multilayers of retinal structure, we developed a photoreceptor/dRPE/PDMS-PmL multilayer device by seeding the iPSC-derived photoreceptor-precursors on the hRPE-coated PDMS-PmL membrane (Figure 6A). The ideal structure of the device was schematically presented in Figure 6B. After co-culturing for 4 weeks, immunofluorescent staining of the bilayer device showed VSX-positive photoreceptor progenitor cells and RPE65-positive RPE cells co-cultured on the PDMS-PmL film (Figure 6C), indicated a successful coating of iPSC/neural progenitor-derived photoreceptor precursor and dRPE cells in our system. Moreover, we investigated the ultrastructure of the iPSC/neural progenitor-derived photoreceptor precursor/dRPE bilayer on the PDMS-PmL film. As shown in Figure 6D, SEM data revealed a monolayer of dRPE on the PDMS-PmL film (Figure 6D, a-b). The three-dimensional retina with photoreceptor precursors was further laid on top of the dRPE monolayer (Figure 6D, c; following step1 and step 2 in Figure 6A). After 4-week of co-culture, the differentiated neural-like and photoreceptor-like cells with typical spiky morphology were observed on the photoreceptor/dRPE/PDMS-PmL multilayer device (Figure 6D, d). These data suggested the capability of PDMS-PmL to carry photoreceptor precursor/RPE multilayer. These data showed the potential of PDMS-PmL bio-scaffold to provide a suitable microenvironment for reconstruction of retinal tissues.

### Validation of long-term biosafety and biostability of PDMS-PmL implant in the subretinal of pigs

**Figure 7:**
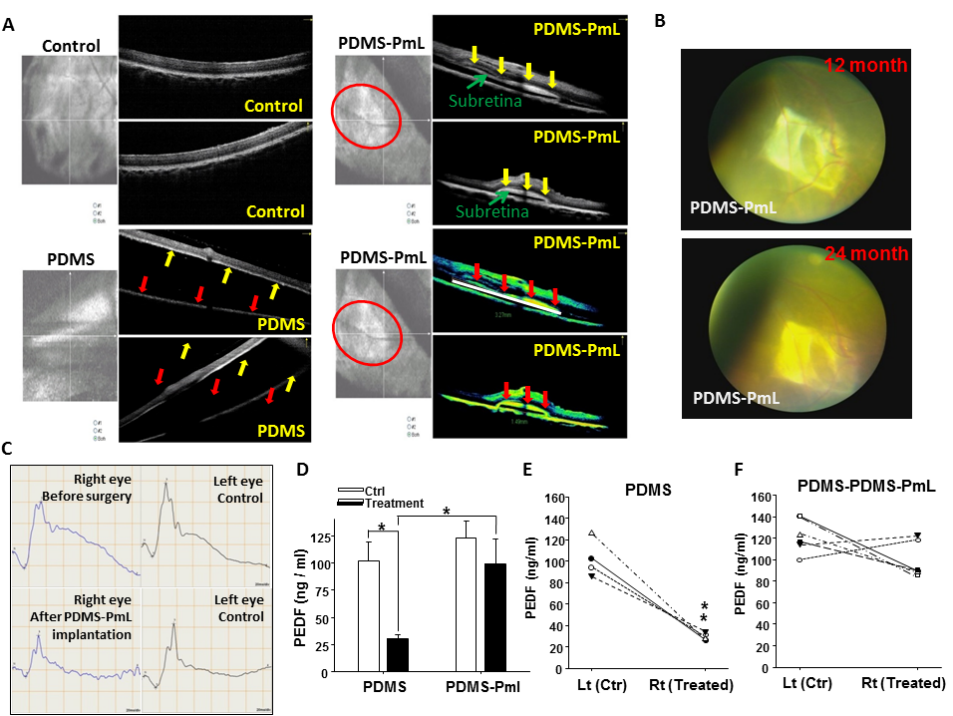
OCT and ocular examination for monitoring long-term biostability of PDMS-PmL in the subretinal space of transplanted porcine **A.** OCT screening demonstrated the well retinal attachment of PDMS-PmL in the subretinal space of transplanted pigs two years after transplantation with retinal surgery. Control: normal retinal without surgery. PDMS: transplantation of non-modified PDMS in the subretinal space of pigs. The gap between yellow and red cursors represents retinal detachment. **B.** Serial observation of funduscopic photography at 12- and 24-month showed that PDMS-PmL implants at the same anatomical position without inducing any complication after transplantation. **C.** Two years after implantation, scotopic ERG responses recorded in PDMS-PmL transplanted eyes were no significantly different from that recorded in control eyes. **D.**-**E.** The macula area of PDMS (*n* = 4) and PDMS-PmL (*n* = 6) eyes, as well as non-surgery control eyes, were isolated 2 years after transplantation, and subjected to ELISA assay for detecting PEDF levels. The mean PEDF levels were showed in the bar chart in **D.**, and the paring control left eyes with treated right eyes were showed in **E.** and **F.** Comparing to decreased levels of PEDF in PDMS eyes, PDMS-PmL eyes showed the maintenance of trophic PEDF levels.

To further validate the long-term biosafety and biostablility of the PDMS-PmL implant *in vivo*, we implanted PDMS and PDMS-PmL in the subretinal space around macular area in 4 and 6 porcine eyes, respectively. After the subretinal-transplantation of PDMS and PDMS-PmL, the retinal anatomical structure, function and ocular condition of each subject were routinely monitored every 3 months by optical coherence tomography (OCT), slit-lamp examination, color fundi photography, and full-field and multifocal electroretinograms (ERGs). In all PDMS and PDMS-PmL-transplanted eyes, the implants were *in situ* and stable without movement. However, in PDMS eyes, the cross-sectional OCT imaging identified disruption and loos of host photoreceptor/RPE layer (Figure 7A; Table 1). Retina detachment was found in some cases two years after the transplantation (Figure 7A). On the contrary, in PDMS-PmL eyes, OCT imaging demonstrated that retinal anatomy was well integrated and the films were placed successfully and maintained stably in the subretinal space of pigs after two-year transplantation (Figure 7A; Table 1). It revealed intact photoreceptor /RPE layer with complete retinal attachment over and around the implant without edema or atrophy. The retina and RPE on both sides of the implant look unaffected. Around the PDMS-PmL subretinal implantation area, the retinal vasculature was preserved without signs of fibrosis or atrophy by color fundi photography at 2-year (Figure 7B). Moreover, the results of our survey demonstrated that no inflammatory signs in anterior chamber and vitreous body were detected (Table 1; Suppl. Table 1). Neither did we found other ocular complications including conjunctiva, cornea, anterior chamber, lens, high intraocular pressure, vitreous body, retinal break, retinal detachment, and retinal hemorrhage in the eyes of these six PDMS-PmL-implanted subjects after (Suppl. Table 1). Importantly, after 2-year transplantation, the results of scotopic-ERG recordings revealed the retinal function to light response in PDMS-PmL transplanted eyes were no significantly different from that recorded in the same eye before surgery or the control eyes (Figure 7C). Collectively, these results confirmed the long-term biostability and biosafety of the PDMS-PmL *in vivo*.

**Table 1:** Clinical ophthalmic observation and analysis for the subretinal transplantation of PDMS and PDMS-PmL bioscaffold at post-implantation 6, 12, and 24 months

Group	Post-implantation month	Cornea/Lens by Slit-Lamp examination	IOP (mmHg) by Pneumotonogram	Vitreous Media by Color fundi photography	Implant/Retina features by Color fundi photography	Implant/Retina features by OCT examination
PDMS (n=4)	6	Normal	15.2 ± 2.1	Clear	Implant in situ Retina well-looking	Implant stable Photoreceptor layer mild edema
	12	Normal	14.3 ± 2.3	Clear	Implant in situ Retina whitening/atrophy	Implant stable Photoreceptor/RPE layer partial degenerated
	24	Normal	15.6 ± 1.5	Clear	Implant in situ Retina atrophy	Implant stable Photoreceptor/RPE layer partial loss
PDMS-PmL (n=6)	6	Normal	15.1 ± 1.7	Clear	Implant in situ Retina well-looking	Implant stable Photoreceptor/RPE layer intact
	12	Normal	15.7 ± 1.9	Clear	Implant in situ Retina well-looking	Implant stable Photoreceptor/RPE layer intact
	24	Normal	15.3 ± 1.8	Clear	Implant in situ Retina well-looking	Implant stable Photoreceptor/RPE layer intact

PDMS (n = 4 pigs); PDMS-PmL (n = 6 pigs)

**Figure 8:**
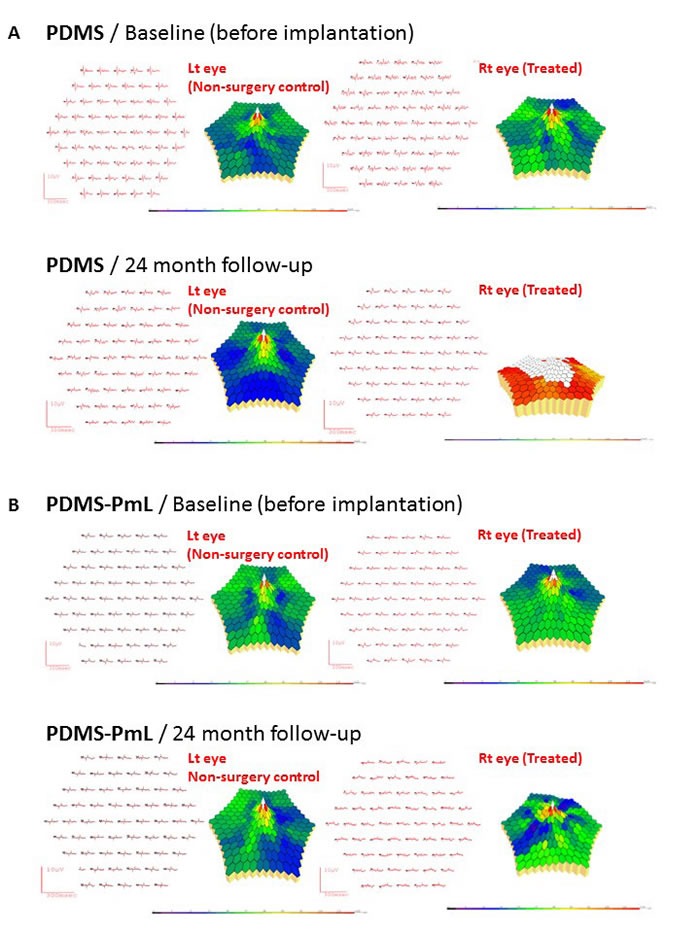
Multifocal ERG recordings for monitoring long-term function of PDMS and PDMS-PmL in the subretinal space of transplanted porcine For each individual mfEGR recording, the right panel shows 3D-topographical map and the left panel shows the trace array. **A.** After 2-year PDMS transplantation in right eye, mfERG recordings were marked depressed comparing to baseline right eye before surgery. The left eye served as a control. **B.** After 2-year PDMS-PmL transplantation in right eye, mfERG recordings showed no significant change comparing to baseline right eye before surgery. The left eye served as a control.

Retinal macula is located in the center of retina and responsible for central, high-resolution vision. However, it is still an open question to precisely measure the macular function of patients with retinal degeneration after stem cell transplantation or bionic retinal-implants. Multi-focal ERG (mtERG) provides an objective approach to analyze the local electrophysiological light-responses, including macular region, in AMD patients as well as in large animals [33-35]. Using mtERG as a platform to evaluate the macular function, right PDMS-transplanted eyes revealed partial depression of mtERG traces at 2-year, suggesting PDMS-related retinal injury (Figure 8A). On the other hand, mfERG signals in PDMS-PmL eyes were preserved generally at 2-year follow-up (Figure 8B). PDMS-PmL transplantation up to 2 years maintained macula function and provide a good microenvironment for subreinal membranous scaffolds. We also isolated the macula area to detect the PEDF levels in PDMS and PDMS-PmL-transplanted eyes. Comparing to decreased levels of PEDF in PDMS eyes, PDMS-PmL eyes showed the maintenance of trophic PEDF levels, indicating PDMS-PmL preserved retinal microenvironment in eyes up to 2 year (Figure 7D-7F). Taken together, these results indicated that PDMS-PmL is able to be implanted in subretinal space without causing severe damages in host's RPE morphology and functions, and also maintain the retinal microenvironment of transplanted subjects. These analyses demonstrated the potential application of PDMS-PmL transplantation for rescuing macular degeneration *in vivo*.

## DISCUSSION

Pigment epithelium-derived factor (PEDF), a neurotrophic protein of the retina, is a member of the serine protease inhibitor superfamily found in the interphotoreceptor matrix in intimate contact with photoreceptors [36, 37]. PEDF prevents apoptosis and enhances the survival of neurons, protects photoreceptors and RPE from degeneration, as well as prevents neovascularization in retina [38, 39]. The plasma PEDF level of AMD patients is significantly decreased and the secretion of PEDF in the conditioned medium from the *in vitro* culture of AMD donor eyes was decreased as well [40, 41], indicating the importance of PEDF in AMD pathogenesis. The RPE cell possess its unique functions in retina, where it carries out phagocytosis to engulf the outer segment of photoreceptors and secrets PEDF to support the growth of photoreceptors, prevent the degeneration of photoreceptor and RPE, and inhibit the neovascularization in retina [36]. Nevertheless, these functions largely depend on the organization of polarized RPE cells lay out as a monolayer *in vivo* [24, 42]. Previous study done by Stanzel *et al.* demonstrated the advantages of polarized RPE monolayer in transplanted rabbit subretinal space [24, 42]. The correct cell organization of RPE largely depends on the integrity of Bruch's membrane, a unique structure located between the RPE and the fenestrated choroidal capillaries of the eye [43, 44]. Here we developed a laminin-coating PDMS biomimetic film, mimicking the Brush's membrane-like subretinal environment, to carry functional differentiated RPE monolayer cell sheet for subretinal implantation. We clearly demonstrated in our SEM and TEM studies that the dRPE cells formed a hexagonally packed monolayer with cellular melanosome deposit on the PDMS-PmL biomimetic film. This dRPE-monolayer on PDMS-PmL expressed prominent microvilli at the apical surface of iPSC-RPEs with typical tight junction. Notably, PDMS-PmL not only facilitated iPSC differentiation toward RPE and accelerated the maturation of polarized dRPE monolayer, but also enhanced the PEDF secretion and phagocytosis function of dRPE cells. These data indicated that the PDMS-PmL biomimetic film provides a Bruch's membrane-like subretinal environment to promote functional maturation of dRPE as a monolayer of cells. mfERG recordings further demonstrated well-preserved macular function in PDMS-PmL transplanted porcine eyes. Individual components of mfERG responses were correlated to non-modified PDMS-induced retinal damage. We establish functional mfERG with simultaneous color fundi photography and OCT imaging for implant transplantation in the porcine eyes. mfERG may be a useful tool for evaluating the macular or local retinal function following an implant insult. Our results showed that this sensitive modality is more suitable for assessing the localized retinal dysfunction after transplantation, and is better than scotopic ERG recordings to detect the functional change at implantation sites to differentiate between a localized functional loss and overall retinal change.

PDMS is a non-degradable material with excellent biocompatibility that can remain bio-stability in patient's lifetime [45]. Compared with the synthetic polymeric scaffolds that have been proposed for subretinal implantation in previous researches [46, 47], PDMS possesses desired physical properties including transparent optical properties, flexible mechanical properties and high oxygen permeability [48], and has been widely used in ocular medical devices like intraocular lenses and contact lenses [49]. However, the surface of PDMS is not easy for cell to attach and long-term proliferate. Through modifying the PDMS film by O_2_ plasma treatment, we conjugated laminin on PDMS surface to enhance the attachment and functional maturation of the dRPE cells attached on it. Physical property analyses showed that this modification did not alter PDMS elastic modulus and permeability, but enhanced its hydrophilic and the cell attachment. Cytotoxicity examination showed that dRPE cells were able to survive and proliferate on the surface of modified PDMS. Our data demonstrated that the plasma treatment and laminin coating did not damage the desired biocompatible properties of PDMS and yet enhanced the adhesion at its interface with dRPE cells. A long-term observation in porcine model confirmed the biostability and efficacy of the modified PDMS film *in vivo* when implanted in subretinal space of pigs (Figure 7 and Figure 8). Moreover, the PDMS-PmL implants did not affect the original light response and preserved the macular function of the implanted animals after 2-year followed up. Notably, we showed in our system that PDMS-PmL is able to carry dRPE/photoreceptor bilayer retinal cells, and both layers retained the morphology and 3D-retinal structure. These data raised the potential clinical applications of the PDMS-PmL, and supported the RPE/photoreceptor coated PDMS-PmL device with multilayer retina-like tissues may be used for treating the end-stage of retinal diseases. Therefore, this PDMS with plasma and laminin modifications would be the transplantable system to deliver the multilayer functional tissues in other clinical surgical implantation for other organ tissues engineering and repairmen.

Taken together, we demonstrated a plasma treated and laminin coated PDMS film that can enhance the attachment, sustain the survival, and facilitate the functional maturation of RPE cells seeded on it. The dRPE/PDMS-PmL implant was able to enhance the response to light stimuli *in vivo*, maintaining the visual-electrophysiological function in transplanted macular retina. Moreover, PDMS-PmL carrying a combination of RPE and photoreceptor precursor cells would be a solution for late-stage AMD. Our findings provide the pre-clinical examinations for the prospective clinical application of pluripotent stem cell-derived retinal-lineage cells/PDMS-PmL subretinal implant in treating retinal degeneration diseases like AMD.

**Figure 9:**
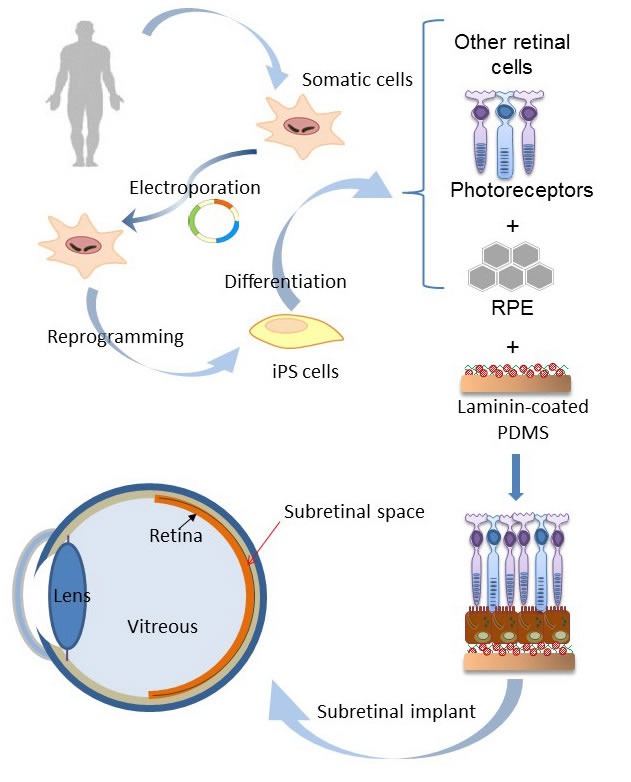
Schematic flow-chart of the potential application of dRPE/PDMS-PmL device in AMD patients

## MATERIALS AND METHODS

### Phagocytosis assay

Phagocytosis is assessed by a flow cytometry-based method using pHrodoTM E. coli fluorescent bioparticles (Invitrogen) which fluoresce when internalized in the reduced pH environment of intracellular phagosomes. Bioparticles do not fluoresce at neutral pH, therefore background fluorescence related to nonspecific adherence is negligible. Bioparticles were prepared as the concentration of 5 μg/μL in Live Cell Imaging Solution (Invitrogen) according to the manufacturer's instructions. Confluent RPE were incubated with 70μL bioparticles plus 630μL HBSS per one well of a 12-well plate in CO2 -independent medium (Invitrogen) for 17-18 hours at 37 °C. Negative control plates were incubated at 4°C. Cells were examined under the microscope, harvested by TrpLE and analyzed by flow cytometry counting 20,000 events on a Flow Cytometer. Positive uptake by phagocytosis is indicated by a rightward shift in fluorescence intensity on histogram plots of the gated cell population.

### PDMS surface modification

The process of PDMS surface modification is consisted with three steps as follow: 1) PDMS oxidation *via* plasma treatment (PDMS-OH). The samples were exposed to oxygen plasma (PC150, JunSun Tech Co., Ltd) to create a hydrophilic surface. They were treated with oxygen plasma at 10^−2^ Torr for 5 mins at 50W, and an oxygen flow rate of 17 sccm. 2) Aminization of PDMS substrates (PDMS-NH2). After plasma treatment, the PDMS membranes were immersed in a silane solutions of 1% by volume APTES (Ca.440140, Sigma-Aldrich, Mo) in absolute ethanol. Then, 5% of by volume DI water was added to the solution to hydrolyze the silane and allowed to react for 15 min at 75°C. The PDMS samples were washed once with 75% by volume aqueous ethanol and the three times with DI water following the silane reaction to remove residual silane compounds. The aminized PDMS membrane is denoted as PDMS-NH_2_. 3) Surface grafting of laminin onto PDMS-NH2 membrane. Conjugation of laminin on PDMS-NH_2_ membrane was performed by crosslinker EDC/NHS (Sigma-Aldrich, Mo). EDC/NHS (1:1 molar ratio) were added to 10 μg/ml laminin in PBS buffer to obtain a final concentration of 10mM, and allowed to react with PDMS-NH_2_ membrane for 1 hr at 37°C. The PDMS membranes were then washed by DI water to remove residual reagents, and rinsed by PBS before the cell seeding.

### Contact angle measurement

Water contact angle on PDMS surfaces were measured at ambient temperature by a video-image sessile drop tensiometer (Model 100SB, Sindatek Instruments Co., Ltd). A 1.5 μl drop of DI water was dropped on the substrate surface and photographed. The shape of the drop and baseline was then fitting by conic section analysis to calculate the three phase (solid-liquid-gas) contact point. For each PDMS substrate, the measurements were performed on five different areas of the surface and the values were averaged.

### Determination of amine content on surface by colorimetric assays

The amount of exposed amine on the silanized PDMS surface (PDMS-NH_2_) was quantified using a colorimetric method— Acid Orange II assay. In brief, aminized PDMS samples (3.9 cm^2^ in 12 well culture dish) were immersed in 1 mL of acid orange dye solution (500 μM) in acid condition (Milli-Q water adjusted to pH 3 by 6N HCl) overnight at room temperature. The samples were then washed 3 times using the acidic solution (pH3) to remove unbound dye. After that, the colored samples were immersed in 1mL of alkaline solution (Milli-Q water adjusted to pH 12 by 6N NaOH) overnight to allow the bound dye on substrates to detach. The amount of the bound dye, representing the amount of surface accessible amine, was quantified by measuring the optical density at 492nm. Different concentration of Acid Orange II solution (10-50μM) were prepared in Milli-Q water and adjusted to pH 12 to establish the standard curve. Unmodified PDMS substrate served as negative control.

### Implantation of dRPE/PDMS-PmL in porcine study

All experimental animals were raised in the Animal Center of Taipei Veterans General Hospital and all surgical procedures were done in accordance with the institutional animal welfare guidelines of National Laboratory Animal Center. Porcine aged between 10 to 12 weeks (around 25 to 30 kg) were chose and operated for checking the long-term biosafety of implants. General anesthesia with isoflurane and endotracheal intubation was performed during surgery [50]. After a sterile procedure by iodine, 23 G vitrectomy (VersaVIT 2.0, Syndergetics) was performed to remove vitreous in the treated eye [51]. A retinotomy was made using intraocular diathermy, followed by injection of viscous fluid to induce an iatrogenic retinal detachment, and the preloaded PDMS-PmL film was delivered precisely into the generated space through a 20-gauge cannula after enlarging the sclerotomy. The flexible scaffold implant then extended spontaneously beneath the photoreceptors in the treated area. After the implantation was ensured, the retina was then reattached with fluid air exchange followed by silicon oil- tamponade. Surgical wounds were ultimately closed by 7-0 vicryl sutures and subconjunctival injection of dexamethasone 4mg/ml and gentamicin 40mg/ml was done immediately after the surgery. Visualized color fundus, OCT images, and ERG for functional determination in all studies were followed and recorded.

### Full-field ERG and multifocal ERG recordings in pigs

The animals were dark adapted for at least a 24-h period overnight, and ERGs

were recorded 12 months and 24 months after transplantation, as described previously with modification [35]. Briefly, the animals were anesthetized with intramuscular injections of 15 mg/kg ketamine/2 mg/kg lidocaine, Anesthesia was maintained with 0.02 μg/kg fentanyl. Mechanical ventilation was established in the volume-controlled mode. The corneas were anesthetized with a drop of 0.5% proparacaine hydrochloride, and the pupils were dilated with 1% tropicamide. For full-field ERG recording, the active contact lens electrodes were placed on the cornea. Responses were amplified differentially, light pulses of 800 cds/m2, bandpass filtered at 0.3-500 Hz, digitized at 0.25- to 0.5-ms intervals by a commercial system (RETIport ERG laptop version, Acrivet, Germany), and stored for processing. The amplitude of the a-wave was measured from the baseline to the trough of the a-wave, and b-wave amplitude was determined from the trough of the a-wave to the peak of the b-wave. The implicit times of the a- and b-waves were measured from the onset of stimuli to the peak of each wave. For multifocal ERG recording, the active contact lens electrodes were placed on the cornea lubricated with Methocel (2% hydroxypropyl-methylcellulose). A ground electrode needle was inserted into the skin behind the ear. The recordings of trace array and 3D recordings were made using UTAS-E3000 system (LKC Technologies, Inc, Gaithersburg, MD). The signal gain was 100,000 and the filter range 3 to 300 Hz with no additional notch filtering.

### Statistical analyses

Results are expressed as mean ± SD. Differences between the groups were analyzed using one-way ANOVA followed by Student's t test. A *P*-value < 0.05 was considered statistically significant.

## SUPPLEMENTARY MATERIAL TABLES


