# Supplementary material for: Laminin modification subretinal bio-scaffold remodels retinal pigment epithelium-driven microenvironment in vitro and in vivo
Source: Oncotarget. 2016 Aug 22;7(40):64631–48. doi: 10.18632/oncotarget.11502 (PMC5323104; doi:10.18632/oncotarget.11502)
Supplement: Supplementary file 1 [file oncotarget-07-64631-s001.pdf]

Laminin modification subretinal bio-scaffold remodels retinal pigment epithelium-driven microenvironment *in vitro* and *in vivo*

Supplementary Material

MATERIALS AND METHODS

Human iPSC generation and culture

Briefly, to generate integration-free iPSCs, cells were nucleofected with 3μg expression plasmid mixture using Amaxa™ human T Cell Nucleofector™ Kit (Lonza). In each nucleofection, 0.83μg PCXLE-hOCT3/4-shp53, 0.83μg PCXLE-hSK, 0.83μg pCXLE-hUL, and 0.5μg pCXWB-EBNA1 were used. 2×10^6 cells were nucleofected with Amaxa Nucleofector II using program V-024 [1]. Cells were cultured the exactly same way as for reprogramming with lentiviral vector expect that every 10–14 days, freshly thawed inactivated mouse embryonic fibroblasts (MEFs) feeder cells were added into each well. The number of ALP-positive iPSC colonies was counted at 3 - 4 weeks after nucleofection. Undifferentiated iPSCs were maintained on inactivated MEFs (50,000 cells/cm2) in human ESC medium (DMEM/F12 (Gibco) supplemented with 20% KnockOut serum replacer (KSR; Invitrogen), 0.1mM non-essential amino acids (Invitrogen), 1mM L-glutamine, 0.1mM s-mercaptoethanol, 10 ng / ml recombinant human basic fibroblast growth factor (bFGF), and antibiotics (Gibco). To prevent cell contamination by MEFs, these iPSCs were transferred to feeder-free/serum-free culture in HESF V2 medium (Cell Science & Technology) without KSR supplementation as described previously [2].

Differentiation of iPSCs into retinal pigment epithelial cells

This research followed the tenets of the Declaration of Helsinki, and the protocols and procedures were approved by the board of the Taipei Veterans General Hospital. All samples were obtained after patients had given informed consent. iPSCs were created from the T cells isolated from patients' peripheral blood (10 ml). The iPSCs were differentiated to RPE according to Dr. Osakada previously established protocol [3]. The iPSC clumps were first incubated in human ES cell culture medium supplemented with 10 μM Y-27632 (WAKO), 5 μM SB431542 (Sigma–Aldrich) and 3 μM CKI-7 (Sigma–Aldrich) for 1 day. The cells were incubated in a differentiation medium (Glasgow minimum essential medium [GMEM; Invitrogen], 1 mM sodium pyruvate, 0.1 mM non-essential amino acids, and 0.1 mM 2-mercaptoethanol) containing 20% knockout

serum replacement (KSR; Invitrogen) for 4 days, then in 15% KSR-containing differentiation medium for 6 days, and finally in 10% KSR-containing differentiation medium for 11–40 days. Y-27632 (10 μM), SB431542 (5 μM) and CKI-7 (3 μM) were added to the differentiation medium for the first 13 and 19 days, respectively. Partially differentiated cells were dissociated and incubated on non-adhesive dishes (Corning) in RPE maintenance medium (DMEM:F12 [7:3] supplemented with B-27 supplement [Invitrogen] and 2 mM l-glutamine [Invitrogen]) for 10 days. The resulting RPE cell aggregates were isolated and replated on CELLstart- (Invitrogen) coated dishes in RPE maintenance medium supplemented with 0.5 μM SB431542 and 10 ng/ml bFGF. The medium was changed every 2–3 days. Thereafter, RPE cells form compact monolayers and re-pigment, typically 90–120 days.

hiPSCs differentiated into RPE cells on PDMS

iPSCs were subcultured onto laminin coated PDMS and maintained in mTeSR1 medium in a feeder-free manner. Before RPE differentiation, the confluence of iPSCs was suggested to reach almost 80C%. From day 1, cells were begun to incubated in RPE differentiation medium, DMEM/F12 (1:1) with 1x B27, 1x N2 and 1x NEAA (non-essential amino acids). Meanwhile, 10 ng/ml IGF-1, 50 ng/ml Noggin and 10 ng/ml Dkk-1 were first introduced into cells while Dkk1 was replaced with 5 ng/ml bFGF after two days later. From day 6 afterwards, 100 ng/ml Activin A and 10μM SU5402 were added and become the major additional stimulators until pigments occurrence. The patches of pigments were able to be observed around day 10. Until numerous pigments appearance, patches of pigments were mechanically picked up followed by gently digestion via TrypLE for 10 min onto another geltrex coated dishes, cultivated in DMEM with 1% FBS.

Cell viability

The influence of PDMS on the cell viability of RPE was evaluated by MTT assay ((3-(4,5-Dimethylthiazol-2-yl)-2,5-diphenyltetrazolium bromide, a yellow tetrazole)). RPE cells were seeded at a density 2x105 cells per well in a 12 well plate with laminin coated PDMS and then incubated in 37 ℃ for 24 to 72 hrs. At various time point, MTT 5 ug/ml was added into per well followed by a further incubation in 37 ℃ for two hours. The MTT contained medium would be then removed and DMSO 1 ml per well was added to dissolve the purple formazan. With a vigorous pipetteing, the well-disturbed dissolved formazan was taken out 100 μl per well into a 96 well plate to determine the absorption values at O.D. 570.

Immunofluorescence staining

The living cells and spheres were fixed in 4% paraformaldehyde, permeabilized in 0.1% Triton X-100, and blocked in 5% normal goat serum- PBS. Cells were incubated with primary antibodies. After washed thrice in PBS, the cells were then incubated with goat anti-mouse or secondary antibodies conjugated with FITC (green) or PE (red). DAPI was used as nuclear stain (blue). Images were obtained using fluorescent microscopy and a digital camera. A list of antibodies and their working conditions are shown as Suppl. Table 2.

Immunohistochemistry

Tissue specimens of patients with FD were collected and retrieved from the archives of the Department of Pathology of Taipei Veterans General Hospital, Taipei, Taiwan. The National Health Insurance (NHI) program in Taiwan, launched in 1995, has successfully provided comprehensive health care for all citizens. Dominant IVS4 mutation and cardiac involvement was predominantly found in Taiwan population. Since the enzyme replacement therapy (ERT) for FD with IVS4 mutation was only approved by Taiwan NHI using cardiac biopsy to confirm FD with cardiomyopathy, all FD cases enrolled in our study were confirmed by myocardium biopsy. The samples were collected using methods that conformed to the ethical guidelines of the Institutional Review Board. This study protocol was approved by the Institutional Review Board of Taipei Veterans General Hospital. A tissue array with heart tumor tissue and normal heart tissue (T301; Biomax, Inc., Rockville, MD) was used as the control group. Immunohistochemistry was performed on 4-mm-thick paraffin-embedded sections of rectal specimens. After deparaffinization and dehydration, the specimens were boiled in 10 mM sodium citrate buffer (pH 6.0) for 40 minutes for antigen retrieval and then blocked in peroxidase-blocking solution (Dako Cytomation, Glostrup, Denmark). The primary antibody (listed in Suppl. Table 2) was incubated at 4°C overnight, and staining was detected using an Envision detection system (Envision detection system, peroxidase/DAB+, rabbit/mouse, Dako Cytomation). The specimens were counterstained with Mayer’s hematoxylin.

Fabrication of PDMS membrane

Polydimethylsiloxane (PDMS) membranes were generated using a medical grade silicone elastomer kit (Sylgard184, Dow Corning, MI.). The PDMS used in the present work is a liquid bi-component silicone pre-polymer, consisting of a base elastomer and a curing agent. The liquid silicone mixture was coated onto tissue culture polystyrene

petri dishes (TCP, Falcon) and allowed to cure in incubator. The membrane rigidity can be controlled by the concentration of cross-linker agent in the PDMS solution (2% ~ 10% ratio of curing agent/base elastomer). After mixing the PDMS, the liquid was degassed in vacuum for 30 min to remove the air bubbles. The liquid mixture silicone was coated onto tissue culture polystyrene petri dishes (TCP, Falcon) and allowed to cure in incubator (65 ^oC) for 90 min. The PDMS membranes were kept in incubator (65 ^oC) for 2 weeks to remove uncured prepolymer from the bulk through thermal aging[4]. The PDMS samples were specially cut into small pieces of ca. 0.05 mm^2 area (0.2 x 0.25 mm) for animal implantation and were sterilized by autoclave before the experiments.

FTIR measurement

The surface composition analysis of functionalized PDMS was characterized by using a grazing angle reflectance (80°) IR measurement. The spectra of samples were recorded on a Fourier Transform Infrared Spectroscopy (Nicolet 6700, Thermo Fisher Scientific, USA). The data were collected in the range of 4000-650 cm^{-1} with 1024 scans at 4 cm^{-1} resolution of images and normalized against the background

Protein quantification on surface via immunochemistry

The amount of attached protein on the modified PDMS surface (PDMS-PmL) was quantified via immunochemistry method. Surfaces modified with laminin were firstly immersed in the blocking buffer (3% Bovine Serum Albumin in PBS buffer) for 1 hr at room temperature. After washing, the substrates were labeled with the anti-laminin, and then stained with fluorescein labeled goat anti-rabbit IgG as the secondary antibody. The relative amount of protein on surface was measured using a microplate reader (TECAN infinite M1000) at an excitation wavelength of 488nm and an emission wavelength of 515nm. Negative controls were performed with the antibodies on unmodified PDMS membrane.

Modified electrochemical spectroscopy:

All electrochemical measurements were performed with a modified dual cells system with connecting pipe. The Au electrode (working electrode) was placed in one of the reaction cell and a coiled platinum wire (counter electrode), and an Ag/AgCl wire immersed in 3 M KCl (reference electrode) were placed in another cell. The membranes to be tested, were placed in the center of connecting pipe. The cells and pipe were filled with PBS buffer. All experiments were performed at room temperature, and all potentials in this work were measured with direct relevance to the Ag/AgCl

reference electrode. The cyclic voltammetry operated from -0.2V to +0.6V (vs Ag/AgCl) and the response currents were recorded by our homemade electrical chemical spectroscopy [5, 6].

Combining dRPE with laminin-coated PDMS

Prior to plate RPE cells onto PDMS, 10 μg/ml laminin was added onto PDMS and incubated at 37℃ for at least 1 hr. Afterwards, isolated P1 RPE cells were then sub-cultured for one time by 0.1 % trypsin to become P2 and $6x10^4$ P2 RPE /cm^2 was transferred to laminin coated PDMS for a following long-term maintenance.

Scanning electron microscopy (SEM)

For observing the morphology of plasma treated PDMS, samples were dried in vacuum and then sputter coated with gold (JFC 1200, JOEL Tokyo, Japan). The SEM image was obtained by FEI Inspect F50 (Hillsboro, OR, USA). To prevent the residual water and fasten the structure, the film was first put into the cell filled with liquid Nitrogen for drying. Then, the film was coated with gold to avoid the accumulation of electrostatic charge at the PDMS surface. The surface topography can be therefore obtained from SEM image with voltage of 30keV. For observing the morphology of dRPE monolayer on modified PDMS, dRPE seeded PDMS samples were maintained in culture to reach confluence within two weeks. The samples were fixed in 2.5% glutaraldehyde / 2% paraformaldehyde in 0.1 M PBS, followed by post-fixing in 1% osmium tetroxide, dehydration through a serious of graded ethanol steps to 100% ethanol, critical point dried (Tousimis AutoSamdri-814, Rockville, MD), and then sputter coated with gold (JFC 1200, JOEL Tokyo, Japan). Images were obtained using a JSM-7600F (JOEL, Tokyo, Japan) scanning electron microscope (SEM) with electron beam energy of 5kV.

Transmission electron microscopy (TEM)

Cells (1×10^7) were suspended in 1.2% agarose and fixed in 0.1 M phosphate buffer (PB), pH 7.4, containing 4% paraformaldehyde and 2.5% glutaraldehyde at 4°C overnight. The samples were washed with 0.1 M PB before post-fixation with 1% OsO4 in 0.05 M PB for 1 hour. After washing with distilled water, the samples were rinsed in block-stain with 0.2% uranyl acetate at 4°C overnight. The samples were dehydrated in a serial dilution of ethanol for 10 min each (from 50% to 100% ethanol) and further infiltrated with a 100% ethanol/acetone (11) mixture and 100% acetone for 15 min each. Then, they were infiltrated with 100% acetone/Spurr resin (11) and (1:3) mixture for 1 hour each. The samples were changed to Spurr resin for continuous

infiltration for 24 hours before being transferred to a capsule filled with Spurr resin. The Spurr resin was polymerized and solidified at 72°C for 48 hours. The resin blocks were trimmed and cut using an ultramicrotome (Leica Ultracut R, Vienna, Austria). Thin sections were transferred to 200 mesh copper grids and stained with 2% uranyl acetate for 20 min and 2.66% lead citrate for 5 min prior to observation with a JEM1400 electron microscope (JEOL USA, Inc., Massachusetts, USA) at 100–120 kV.

SUPPLEMENTAL TABLES

Supplemental Table 1. Examination protocols and adverse events in 6 pigs with PDMS-PmL implantation for up to 2 years.

The follow-up OCT and ocular examination for validating the condition of 6 PDMS-PmL implanted subjects. No ocular complications were noted in these 6 subjects.

Follow up time		6 months						12 months						18 months						24 months (2 year)					
Numbers		1	2	3	4	5	6	1	2	3	4	5	6	1	2	3	4	5	6	1	2	3	4	5	6
Slit-lamp & Pneumotonogram	Conjunctiva	N	N	N	N	N	N	N	N	N	N	N	N	N	N	N	N	N	N	N	N	N	N	N	N
	Cornea	N	N	N	N	N	N	N	N	N	N	N	N	N	N	N	N	N	N	N	N	N	N	N	N
	Anterior Chamber	N	N	N	N	N	N	N	N	N	N	N	N	N	N	N	N	N	N	N	N	N	N	N	N
	Lens	N	N	N	N	N	N	N	N	N	N	N	N	N	N	N	N	N	N	N	N	N	N	N	N
	Intraocular Pressure	NR	NR	NR	NR	NR	NR	NR	NR	NR	NR	NR	NR	NR	NR	NR	NR	NR	NR	NR	NR	NR	NR	NR	NR
OCT & fundus photography	Vitreous Body	N	N	N	N	N	N	N	N	N	N	N	N	N	N	N	N	N	N	N	N	N	N	N	N
	Retinal Break	nil	nil	nil	nil	nil	nil	nil	nil	nil	nil	nil	nil	nil	nil	nil	nil	nil	nil	nil	nil	nil	nil	nil	nil
	Retinal detachment	nil	nil	nil	nil	nil	nil	nil	nil	nil	nil	nil	nil	nil	nil	nil	nil	nil	nil	nil	nil	nil	nil	nil	nil
	Retinal Hemorrhage	nil	nil	nil	nil	nil	nil	nil	nil	nil	nil	nil	nil	nil	nil	nil	nil	nil	nil	nil	nil	nil	nil	nil	nil

N: normal; NR: normal range (intraocular pressure from 10 to 21 mmHg); Nil: no adverse event.

Supplemental Table 2. List of proteins tested by antibodies

Protein	Assay	Antibody	Origin	Dilution	Incubation period
ZO-1	IF	rpab	Thermo, MA, USA	1:100	O.N.
ZO-1	IF	mmab	Thermo, MA, USA	1:200	O.N.
RPE65	IF	mmab	Thermo, MA, USA.	1:250	O.N.
Bestrophin	IF	rpab	Abcam, MA, USA.	1:200	O.N.
MITF	WB	mmab	Abcam, MA, USA.	1:1000	O.N.
MITF	IF	mmab	Abcam, MA, USA.	1:200	O.N.
PAX6	IF	rpab	Thermo, MA, USA	1:50	O.N.
Otx2	WB	rpab	Abcam, MA, USA.	1:500	O.N.
GAPDH	WB	rpab	Ab9385, Abcam, Inc	1:5000	60 min

Abbreviations: IF, immunofluorescence; WB, Western blot; mmab, mouse monoclonal antibody; rpab, rabbit polyclonal antibody; O.N.: overnight.

REFERENCES

1. Okita K, Yamakawa T, Matsumura Y, Sato Y, Amano N, Watanabe A, Goshima N and Yamanaka S. An efficient nonviral method to generate integration-free human-induced pluripotent stem cells from cord blood and peripheral blood cells. Stem Cells. 2013; 31(3):458-466.
2. Chien Y, Liao YW, Liu DM, Lin HL, Chen SJ, Chen HL, Peng CH, Liang CM, Mou CY and Chiou SH. Corneal repair by human corneal keratocyte-reprogrammed iPSCs and amphiphatic carboxymethyl-hexanoyl chitosan hydrogel. Biomaterials. 2012; 33(32):8003-8016.
3. Osakada F, Jin ZB, Hirami Y, Ikeda H, Danjyo T, Watanabe K, Sasai Y and Takahashi M. In vitro differentiation of retinal cells from human pluripotent stem cells by small-molecule induction. Journal of cell science. 2009; 122(Pt 17):3169-3179.
4. Eddington DT, Puccinelli JP and Beebe DJ. Thermal aging and reduced hydrophobic recovery of polydimethylsiloxane. Sensors and Actuators B: Chemical. 2006; 114(1):170-172.
5. Li HY, Tseng SH, Cheng TM, Chu HL, Lu YN, Wang FY, Tsai LY, Shieh JY, Yang JY, Juan CC, Tu LC and Chang CC. Rapid and highly sensitive detection of Enterovirus 71 by using nanogold-enhanced electrochemical impedance spectroscopy. Nanotechnology. 2013; 24(28):285102.
6. Cheng TM, Lee TC, Tseng SH, Chu HL, Pan JP and Chang CC. Human haptoglobin phenotypes and concentration determination by nanogold-enhanced electrochemical impedance spectroscopy. Nanotechnology. 2011; 22(24):245105.
